# Debridement, antibiotics and implant retention (DAIR) is successful in the management of acutely infected unicompartmental knee arthroplasty: a case series

**DOI:** 10.1080/07853890.2023.2179105

**Published:** 2023-02-22

**Authors:** Angela Brivio, Talal Al-Jabri, Jurgen Martin, David Barrett, Nicola Maffulli

**Affiliations:** a Department of Trauma and Orthopedic Surgery, Istituto Clinico Città Studi, Milano, Italy; b King Edward VII’s Hospital, London, United Kingdom; c Trauma and Orthopaedic Surgery, Department of Surgery and Cancer, Imperial College London, United Kingdom; d Vulpius Klinik, Bad Rappenau, Germany; e Spire Hospital, Southampton, United Kingdom; f School of Engineering Sciences, University of Southampton, Highfield, Southampton, United Kingdom; g Department of Medicine, Surgery and Dentistry, University of Salerno, Baronissi, SA, Italy; h Barts and the London School of Medicine and Dentistry, Centre for Sports and Exercise Medicine, Mile End Hospital, Queen Mary University of London, London, United Kingdom; i School of Pharmacy and Bioengineering, Keele University School of Medicine, Stoke on Trent, United Kingdom

**Keywords:** Unicompartmental knee arthroplasty, failure, infection, dair, revision, periprosthetic joint infection

## Abstract

**Background:**

Infections are rare and poorly studied complications of unicompartmental knee arthroplasty (UKA) surgery. They are significantly less common compared to infections after total knee arthroplasties (TKAs). Optimal management of periprosthetic joint infections (PJIs) after a UKA is not clearly defined in the literature. This article presents the results of the largest multicentre clinical study of UKA PJIs treated with Debridement, Antibiotics and Implant Retention (DAIR).

**Materials and Methods:**

In this retrospective case series, patients presenting between January 2016 and December 2019 with early UKA infection were identified at three specialist centres using the Musculoskeletal Infection Society (MSIS) criteria. All patients underwent a standardized treatment protocol consisting of the DAIR procedure and antibiotic therapy comprising two weeks of intravenous (IV) antibiotics followed by six weeks of oral therapy. The main outcome measure was overall survivorship free from reoperation for infection.

**Results:**

A total of 3225 UKAs (2793 (86.2%) medial and 432 (13.8%) lateral UKAs) were performed between January 2016 and December 2019. Nineteen patients had early infections necessitating DAIR. The mean follow-up period was 32.5 months. DAIR showed an overall survivorship free from septic reoperation of 84.2%, with overall survivorship free from all-cause reoperation of 78.95%.

The most common bacteria were Coagulase-negative *Staphylococci*, *Staphylococcus aureus* and Group B *Streptococci*. Three patients required a second DAIR procedure but remained free from re-infection at follow-up obviating the need for more demanding, staged revision surgery.

**Conclusions:**

In infected UKAs, the DAIR procedure produces a high rate of success, with a high survivorship of the implant.

**Key messages**
- Debridement, Antibiotics and Implant Retention (DAIR) is a successful and minimally invasive surgical option for the management of periprosthetic joint infections (PJIs) after UKA.
- The surface area available for bacteria to colonise is much smaller in UKAs compared to total knee arthroplasties (TKAs), and this may account for the higher success rates of the DAIR procedure in infected UKAs versus infected TKAs.
- A second DAIR procedure can be considered in the management of the early recurrence of PJIs with a well-fixed UKA.

## Introduction

Unicompartmental Knee Arthroplasty (UKA) is more commonly performed: the United Kingdom (UK) National Joint Registry (NJR) has now recorded 125,455 UKAs [1]. The registry data show a rapid increase in the volume of UKAs performed annually since 2014, with UKAs accounting for 8.4% of all primary knee replacements in 2014 versus 13% in 2020 (1). UKA functions as a resurfacing prosthesis over the femoral and tibial surface of one compartment of the knee alone, rather than replacing the whole knee as in total knee arthroplasty (TKA). New designs have improved the long-term survivorship of these implants: the 10-year UKA survival rate is now 90% to 95% [2–4]. Compared to TKA, the advantages of UKA include bone stock preservation, more physiological knee kinematics (which translates into better functional outcomes), less blood loss, faster recovery, fewer medical complications (venous thromboembolic and cardiac events) and reduced overall morbidity and mortality rates [2,5,6]. UKA produces satisfactory outcomes in well-selected patients with primary or secondary unicompartmental knee osteoarthritis.

Patients undergoing TKA are twice as likely to develop deep infection compared to UKA [7]. The rate of infection in UKA is substantially lower than in TKA, with an incidence varying from 0.1% to 1.0% [8–10].

Signs and symptoms of UKA infection are comparable to TKA infection, and the diagnostic algorithm is the same [11,12]. UKA infection can have an early or delayed onset, and hematogenous seeding is possible.

Guidelines to address the specific treatment of early periprosthetic joint infection (PJI) in UKAs are limited [13–16], particularly with Debridement, Antibiotics and Implant Retention (DAIR). As reported in the proceedings of the 2018 International Consensus Meeting (ICM) on PJI, DAIR can be considered but, if initial efforts result in failure or if a chronic infection is present, the implanted prosthesis should be removed, and a one-stage or two-stage conversion to TKA should be performed in combination with antibiotic therapy [11]. Therefore, the management of early infection in UKA is described as identical to that of TKA.

Hernandez et al. reported greater success with a two-stage revision arthroplasty (100% at 5 years) versus the DAIR procedure (61% at 5 years) [14]. However, in the recent series by Chalmers et al. from the Hospital for Special Surgery in New York, no significant differences in outcomes were found between a two-stage revision versus the DAIR procedure (16). Interestingly, Chalmers et al. reported that two patients who underwent a DAIR procedure required a second DAIR procedure and were stable without persistent PJI at the final review [16]. One patient in Hernandez et al.’s series also was treated successfully with a repeat DAIR [14].

Following UKA, a significantly lower proportion of foreign or artificial material is implanted in the knee joint. This may explain the lower rate of infection in comparison to TKA. Given the dimensions of the UKA implants compared to a TKA, more native tissue is retained in UKAs, and this should favourably influence the immunological status and natural defences of the operated knee. When considering the increasing numbers of UKAs being performed over the last two decades, the limited studies available to date and the potential for a better natural response to early infection, we believe it is appropriate to re-examine the effectiveness of the DAIR procedure in the management of early infections in UKA [1,17–19]. Accurate diagnosis and DAIR may be the appropriate treatment for early UKA infections, with a higher rate of success and retention of the prosthesis than in TKA.

The aim of this study is to present the results of the largest case series to date of early UKA PJIs treated using the DAIR procedure between January 2016 and December 2019 at three different European institutions showing causative bacteria, surgical treatment, antibiotic therapy and outcome at last follow-up (FU).

## Materials and methods

In this retrospective study, anonymised data from the review of the case notes of all patients undergoing UKA from three different European hospitals were collated. (Vulpius Klinik, Vulpiustrasse 29, 74906, Bad Rappenau (Germany); Southampton University Hospital, Tremona Road, Southampton, SO16 6YD (UK), Istituto Clinico Città Studi, *via* Niccolò Jommelli, 17, 20131, Milano (Italy)).

Patients with UKAs who met the diagnostic criteria for PJI highlighted by the Musculoskeletal Infection Society (MSIS) and modified by the International Consensus Meeting (ICM) in 2013 were included in the study [11,12]. Exclusion criteria consisted of any patient who did not fulfil the Musculoskeletal Infection Society (MSIS) diagnostic criteria for a PJI, those with a primary TKA/revision prosthesis *in situ* and patients who had suffered from septic arthritis of the native knee joint previously (Table 1) [11,12]. Only patients with symptoms of acute PJI with symptoms present for less than four weeks were included in this study. The exact definition of an acute PJI is controversial, with time frames in the literature ranging from 4 weeks postoperatively to 90 days. However, a DAIR procedure is commonly believed to be most successful when performed within the first 4 weeks of the presentation of the infection [18]. Therefore, we defined as acute PJIs those occurring less than 4 weeks from UKA implantation. An acute PJI may also occur secondary to haematogenous seeding of a previously well-functioning joint, and PJIs presenting with symptoms duration of fewer than 4 weeks were also considered to be acute. This study focuses on acute PJIs presenting after primary surgery, and all patients were operated on within eight weeks of the index UKA procedure. Chronic PJIs were defined as those where symptoms had been present for longer than 4 weeks [20,21], and were therefore excluded from the present study.

**Table 1.** Periprosthetic Joint Infection. Details and Treatment.

Patients (*n* = 19)	UKA side	Time from Primary UKA to DAIR (days)	CRP (mg/L)	Organism(s)	Follow-up (months)	Antibiotic(s) administered
1	M	16	52.5	*Staphylococcus aureus*	53	Rifampicin, Cefuroxime, Cotrimoxazole.
2	M	15	73.5	*Staphylococcus epidermidis multi-resistant*	52.5	Cefuroxime, Rifampicin, Ciprofloxacin.
3	M	8	65.5	*Staphylococcus epidermidis multi-resistant*	51	NA
4	L	14	NA	*Enterobacter cloacae*	49.5	Cefuroxime, Ciprofloxacin, Rifampicin.
5	M	12	87	*Staphylococcus aureus*	47	Rifampicin, Cefuroxime, Cotrimoxazole.
6	M	12	243	*Streptococcus mitis*	46	Rifampicin, Ciprofloxacin, Clindamycin.
7	L	50	154	*Staphylococcus epidermidis*	43	Rifampicin, Vancomycin, Flucloxacillin.
8	L	19	2.5	*Streptococcus mitis, Streptococcus oralis*	34.5	Cefuroxime, Rifampicin, Ciprofloxacin, Clindamycin.
9	M	12	95	*Staphylococcus aureus*	33.5	Ciprofloxacin, Rifampicin.
10	M	16	2.5	*Staphylococcus aureus, staphylococcus epidermidis*	28	Cefuroxime, Cotrimoxazole.
11	M	55	89	*Staphylococcus epidermidis*	27.5	Ciprofloxacin, Rifampicin.
12	M	33	119	*Staphylococcus epidermidis, Staphylococcus coagulase negative, Corynebacterium.*	NA	Cefuroxime, Rifampicin, Ciprofloxacin.
13	L	22	25	*Staphylococcus aureus*	24	Cefuroxime, Rifampicin, Amoxicillin/Clavulanic Acid.
14	M	16	171	*Staphylococcus capitis, Staphylococcus epidermidis*	22.5	Rifampicin, Cefuroxime, Ciprofloxacin.
15	M	11	75.9	*Staphylococcus epidermidis*	18	Rifampicin, Vancomycin, Cotrimoxazole.
16	M	22	18.5	*Acinetobacter baumannii complex*	16	Vancomycin, Rifampicin, Ampicillin/Sulbactam, Cotrimoxazole.
17	M	37	111	*Staphylococcus epidermidis*	14	Rifampicin, Flucloxacillin.
18	M	34	58	*Staphylococcus aureus*	13	Rifampicin, Vancomycin, Cotrimoxazole.
19	L	15	233	*Staphylococcus aureus, Enterococcus faecalis*	11.5	Vancomycin, Rifampicin, Amoxicillin/Clavulanic Acid.

CRP: C-Reactive Protein; DAIR: Debridement, Antibiotics and Implant Retention; L: Lateral; M: Medial; NA: Not Available/Applicable; UKA: Uni-compartmental Knee Arthroplasty.

The primary outcome measure was survivorship from infection after DAIR for infected primary UKAs. We reported the total number of UKAs per year, performed between January 2016 and December 2019, and the number of acutely infected UKAs per year in the same period highlighting the compartment with the prosthesis (medial or lateral), the period between the primary surgery and the subsequent DAIR procedure performed for a PJI. We recorded the results of preoperative and intraoperative microbiological cultures. All PJIs treated with DAIR procedures were performed by surgeons with experience in complex knee arthroplasty using a standardized technique. The DAIR procedure consists of a complete synovectomy, removal of the polyethene tibial insert, deep irrigation with 50:50 solution of at least 5 L of sterile 0.9% normal saline and 3% hydrogen peroxide followed by 0.9% normal saline irrigation to ensure satisfactory antimicrobial activity and minimal toxicity to tissues and implant materials. During synovectomy, at least 5 samples for gram stain, microscopy, culture and sensitivity analysis were taken. A new sterile tibial polyethene implant was then inserted and the wound closed. We noted whether more than one operation was performed per patient and the consequent results. The antibiotic therapy per patient was recorded, as were any changes in the choice of antibiotics as a result of secondary cultures.

All patients were assessed by an infectious diseases specialist pre- and post-operatively to tailor organism-specific parenteral antibiotics for two weeks followed by six weeks of oral antibiotics.

Patients failing to complete the therapeutic regimen through non-attendance or failure of therapy were excluded, and recurrence of infection was noted. Failure to eradicate the infection was defined as recurrent infection from the same or different organisms. FU was continued in successful cases until inflammatory markers had returned to normal and the patient reported cessation of infective symptoms. Yearly FU appointments were then scheduled. The overall survivorship free from reoperation for infection and the overall survivorship free from reoperation for any reason is reported. The mean and standard deviations are provided using a population standard deviation formula. We used a Kaplan Meier survivorship curve to present survivorship free from reinfection following the primary DAIR procedure. This mode of analysis is similar to that used in previous literature in this field and allows direct comparison to other previous publications and different techniques for treating PJI.

## Results

**Figure 1.**
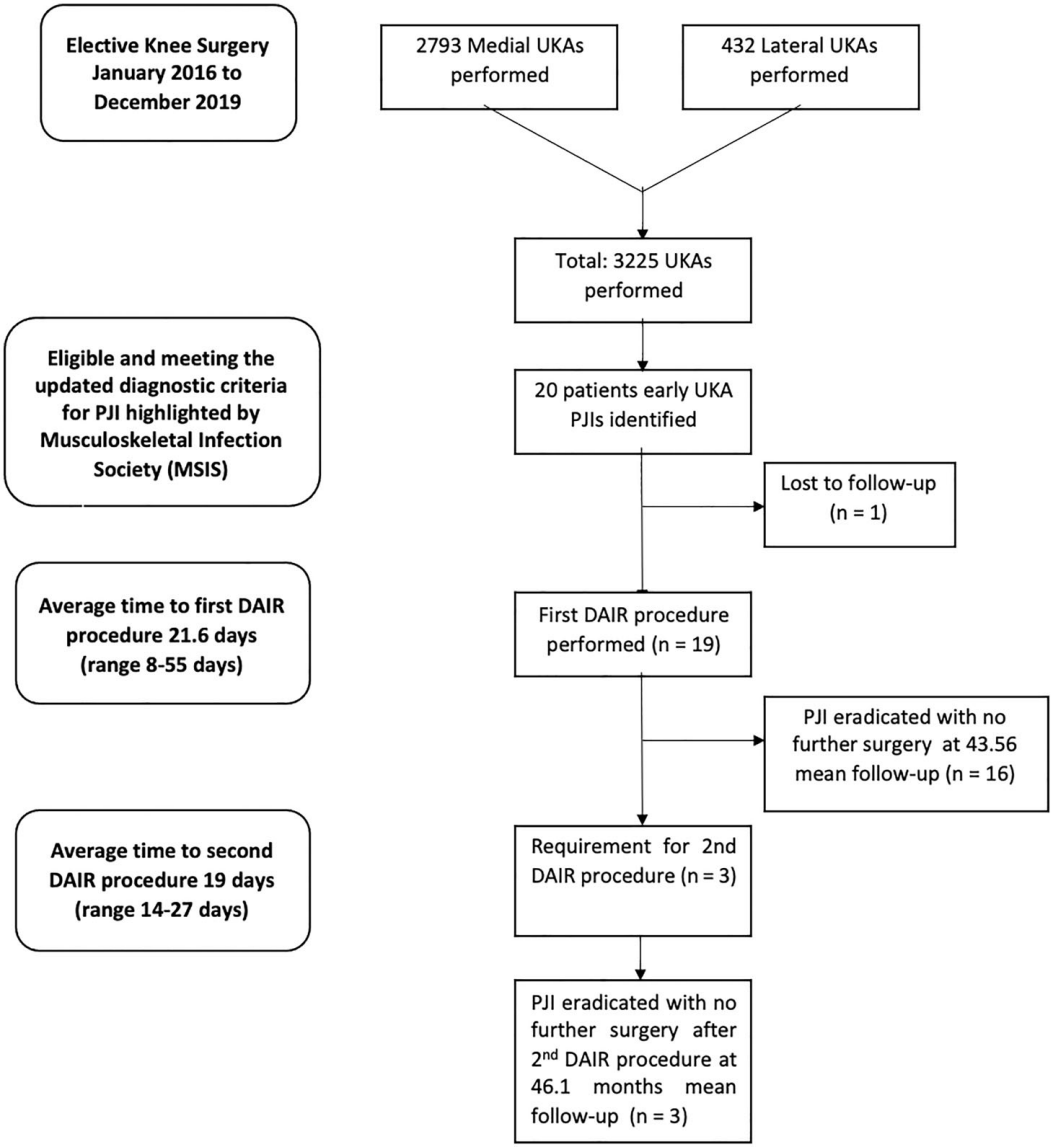
Summary flowchart according to STROBE recommendations illustrating the patients analysed.

**Figure 2.**
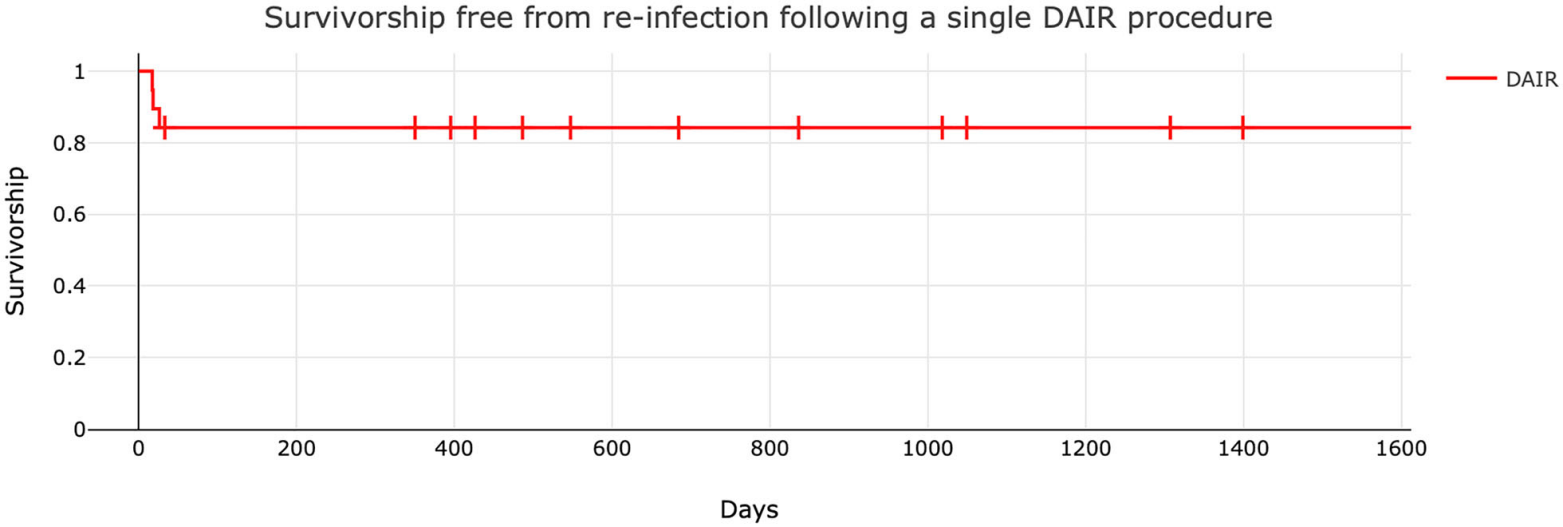
Kaplan–Meier Graph demonstrating survivorship free from re-infection following a single DAIR procedure in acute UKA infections. Each Dash 
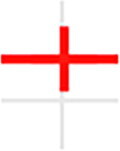
 represents the point of patient discharge from follow-up with the advice to return to the clinic should symptoms recur.

A total of 3225 UKA (2793 (86.2%) medial and 432 (13.8%) lateral UKA) were performed between January 2016 and December 2019. We identified 19 (0.59%) patients diagnosed with acute PJI according to our criteria after UKA (Tables 1–5, Figures 1 and 2).

**Table 2.** Complications after the first DAIR: Details and Treatment.

Patients (*n* = 4)	UKA side	Time from Primary UKA to 1st DAIR (days)	Time from 1st DAIR to 2nd DAIR procedure (days)	Details of 3rd operation	Organism(s)	Follow-up (months)
1	L	15	18	NA	*Enterobacter cloacae*	49.5
2	M	17	26	NA	*Staphylococcus aureus, staphylococcus epidermidis*	28
3	M	33	Planned for arthroscopy for an unrelated meniscal lesion.	NA	*Staphylococcus epidermidis, Staphylococcus coagulase negative, Corynebacterium.*	NA
4	L	22	17	260 days later successful TKA was performed for osteoarthritis progression-free from infection postoperatively.	*Staphylococcus aureus*	24

DAIR: Debridement Antibiotics and Implant Retention; L: Lateral; M: Medial; NA: Not Available/applicable; UKA: Uni-compartmental Knee Arthroplasty.

**Table 3.** Demonstrating the statistical features of all the patients who underwent primary DAIR procedures (*n* = 19).

	Mean	Standard deviation
Age (years)	71	8.03
Time to first DAIR (days)	22.05	13.1
Follow-up (months)	32.5	14.57
C-reactive protein levels (milligrams per Litre)	93	68.2

DAIR: Debridement Antibiotics and Implant Retention.

**Table 4.** Demonstrating the statistical features of patients who underwent a second DAIR procedure (*n* = 3).

	Mean	Standard deviation
Age (years)	66.3	11.12
Time to first DAIR (days)	18	2.83
Time to second DAIR (days)	20.3	4.03
Follow-up (months)	24.8	2.3
C-reactive protein levels (milligrams per Litre)	66.2	74.7

DAIR: Debridement, Antibiotics and Implant Retention.

**Table 5.** Demonstrating the statistical features of all the patients who were successfully treated with a single DAIR procedure (*n* = 16).

	Mean	Standard deviation
Age (years)	71.8	7.0
Time to DAIR (days)	22.8	14
Follow-up (months)	34	15.5
C-reactive protein levels (milligrams per Litre)	98.5	65.6

DAIR: Debridement, Antibiotics and Implant Retention.

The details of the 19 patients are summarised in Table 3. The mean FU of this group was 32.5 months and the mean time from primary surgery to DAIR was 22 days. The average age of the 19 patients was 71 years. Five patients were females and 14 were males; five lateral and 14 medial UKA were infected. There were 14 mobile bearings (cemented Oxford Partial Knee, Zimmer-Biomet, Warsaw, IN, USA) used and five fixed bearings (cemented Sigma Partial Knee DePuy Synthes, Warsaw, IN).

The 3 patients who suffered a recurrent infection (Table 4) and required a second DAIR procedure were younger (average age 66 years) and had lower CRP levels (66 mg/l versus 98 mg/l). This group underwent their second DAIR procedure on average 20 days after the first DAIR procedure. Analysis of the 16 patients who underwent DAIR successfully (Table 5) shows an average age of 72 years, a time to DAIR of 23 days, and an average CRP level of 98 mg/l.

The causative bacteria in our cohort were most commonly multi-resistant *Staphylococcus epidermidis* in nine cases and *Staphylococcus aureus* in seven cases. Other bacteria isolated are detailed in Table 1. A number of patients had more than one causative bacteria (Tables 1 and 2).

Three patients underwent two DAIR procedures. The first patient was a 71-year-old female with an infected medial UKA. Her first DAIR procedure was complicated by wound dehiscence which necessitated a second DAIR procedure 15 days later. She was free from re-infection at long-term FU (49.5 months postoperatively). The isolated bacterium was *Enterobacter cloacae* on both occasions.

The second patient was a 51 years old male with an infected lateral UKA: he received a second DAIR procedure 26 days after the first DAIR. The bacteria isolated at the first DAIR were Staphylococcus aureus and *Staphylococcus epidermidis*. Multi-resistant *Staphylococcus epidermidis* was identified at the second DAIR. This patient remained free from re-infection, and was discharged after 28 months of FU.

The third patient was a 77-year-old female with an infected lateral UKA who underwent a second DAIR 16 days after the first one. The bacterium identified at the first DAIR was Staphylococcus aureus, while a Corynebacterium was isolated at the second DAIR procedure. This patient remained free from re-infection but underwent a TKA 260 days after his last DAIR procedure due to the progression of medial compartment osteoarthritis.

A 79-year-old male patient with an infected UKA had a successful DAIR. The bacteria isolated were *Staphylococcus epidermidis*, coagulase-negative *Staphylococcus*, and Corynebacterium. This patient was also, scheduled for an arthroscopy because of an unrelated meniscal tear planned to occur once the infection had been eradicated but was subsequently lost to FU.

One patient was diagnosed with an undisplaced tibial plateau fracture after 2 months from DAIR following a fall. This healed without the need for a further surgical procedure. Table 2 summarises the characteristics of the patients who underwent more than one surgery.

In summary, there were three recurrences of early infections after the first DAIR procedure with overall survivorship free from septic reoperation of 84.2% at long-term FU. The overall survivorship free from all-cause reoperation was 78.95%. All patients who underwent a second DAIR procedure remained free from re-infection at long-term FU.

## Discussion

In our cohort of patients with an infected UKA, DAIR demonstrated overall survivorship free from reoperation of 84.2%, with an overall survivorship free from all-cause reoperation of 78.95%.

An increasing number of UKA procedures are being performed [1,17]. Whilst PJI after UKA is less common than in TKA, there is little guidance for treatment other than replicating the current treatment for TKA. The literature lacks studies directly evaluating the role of the DAIR procedure in infected UKAs [13–16]. Argenson et al. stated *“in the event of acute infection after UKA, early irrigation and debridement followed by antibiotic administration with implant retention can be considered. However, if initial treatment effort results in failure or chronic infection is present, the implanted prosthesis should be removed and a one-stage or two-stage conversion to TKA should be performed in combination with antibiotic therapy”* [18]. These recommendations are conjectural as only a few published scientific articles examine the outcomes of infected UKAs (Table 6). Hernandez et al. retrospectively reviewed 22 years of Mayo Clinic data [14]. Fifteen patients had a PJI after UKA, 5 patients had an early postoperative infection, 5 had acute hematogenous infections, and 5 had chronic infections. Eleven patients underwent a DAIR procedure and 4 underwent a two-stage revision as the initial treatment. DAIR had a success rate of 61% in comparison to two-stage exchanges (100% success). They concluded that DAIR was not appropriate. However, their study was limited by a number of factors, including the duration of the study is over a 20-year period, during which the diagnosis and management of PJIs had gone through numerous changes. They reported that the decision to perform a DAIR or a two-stage revision was not standardized and there was a limited FU in their small cohort. Additionally, acute and chronic cases were included with variable times elapsed from surgery [14]. Chalmers et al. reported on 21 patients treated for UKA PJI: 16 DAIR procedures were performed (14 were for early postoperative PJIs, 1 was described as an acute hematogenous infection and 1 was a chronic infection) [16]. A success rate of 64% was reported, but again this report mixes chronic and acute infections. Labruyère et al. reported success in 9 infected cases treated with a one-stage revision to TKA. Five of these patients had a previous DAIR [15]. DAIR was successful in 44%.

**Table 6.** Summarising currently published results in addition to this study's.

Author(s) and year of publication	Study type	Number of cases	Acute or chronic infections	Number of DAIR procedures performed	Percentage success
Labruyère et al. [15]	Retrospective	9	mixed	5	44%
Hernandez et al. [14]	Retrospective	15	mixed	11	61%
Chalmers et al. [16]	Retrospective	21	mixed	16	64%
Brivio et al. (current results)	Retrospective	19	acute	19	84%

DAIR: Debridement, Antibiotics and Implant Retention.

When reviewing the limited available literature, a marked heterogeneity between studies (e.g. inclusion of acute and chronic PJIs, time to DAIR surgery, and non-standardised techniques) emerges. This makes any useful comparisons difficult. However, the present study seeks to expand the available body of knowledge and should facilitate future systematic reviews [13,14,16,18]. We report a higher than previously recorded success with DAIR in acutely infected UKA, and a higher rate of success with DAIR than is described with TKA (84% success in our study versus 31-72% for DAIR in TKAs [22–28].

Our study may show a greater level of success because it focuses exclusively on acutely infected cases occurring within four weeks after the index surgery, with strict criteria for inclusion and a modern, standardized technique of debridement, polyethene exchange and specific antibiotic therapy is employed. Conditions and procedures were identical at the three different contributing European centres, where treatment plans were developed by multidisciplinary teams, including microbiologists.

All centres use a solution including hydrogen peroxide to lavage the knee. This was selected to ensure satisfactory antimicrobial activity and minimal tissue and implant toxicity. The commonly used 3% solution of hydrogen peroxide has broad antimicrobial activity *in vitro*, with its greatest effect on gram-positive organisms, a feature especially relevant in this context. There are a number of mechanisms for hydrogen peroxide-mediated antimicrobial activity (e.g. oxidation of proteins, lipids, DNA damage etc) [29–32]. In the presence of orthopaedic implants, peroxide can reduce biofilm formation by bacteria including *Pseudomonas aeruginosa* and *Staphylococcus epidermidis* [31]. Additionally, the effect on implants is minimal [33]. These modern techniques and the selective and controlled nature of this study may explain why the reported success of DAIR is higher than previously recorded.

DAIR is shown to be considerably more effective in UKA than TKA (84% versus 31-72% [22–28]. Initially, it was suggested that PJI after UKA might be difficult to treat with DAIR because the native cartilage in the un-resurfaced compartments of the knee could become infected. If this were indeed the case, the infected surfaces can provide a nidus for ongoing infection, leading to chondrocyte necrosis and potentially hastening the osteoarthritic process of the other knee compartments and recurrence of infection [19]. Hernandez et al. performed extensive debridement of the native articular cartilage, but reported unfavourable outcomes, whereas Chalmers et al. did not report debriding native cartilage [14,16]. The treatment of septic arthritis in the native knee is highly successful with arthroscopic irrigation [19]. In the present study, no debridement of the native cartilage was undertaken, and only extensive lavage was performed. The issue of infection of the remaining articular cartilage appears not to affect the outcome. The increased rate of success in comparison to DAIR in TKA is probably a consequence of the significantly lower surface area of artificial material available for bacterial adherence and biofilm formation. Significantly more natural and functional immunologically competent tissue remains in a UKA: in particular, the synovium may allow a more effective response to infection, once the bacterial load is reduced by DAIR. Delivery and penetrance by antibiotics may also be enhanced by intact native structures with a healthy and undisturbed blood flow. The type and distribution of the causative bacteria in this group of UKA patients are identical to that seen in TKA infections, so this appears not to be a factor.

The treatment of reinfection following DAIR in UKA varies. Conversion to a TKA or two-stage revisions is the common option [14,16]. Secondary DAIR has also been described in small numbers [14,16]. In the present study, each patient who suffered a recurrence underwent a second DAIR with a high rate of success and implant retention. Whilst this is not undertaken in the treatment of TKA infection, the qualities of the small implant surface and retention of a significant part of the native knee may make this a treatment option specific to UKA.

Several limitations to this study are evident. Firstly, its retrospective nature and the lack of a control group. Although the largest single study on the use of the DAIR procedure for early UKA PJIs, we acknowledge that the sample size of the present investigation is relatively small: this makes subgroup analysis for risks of reoperation unfeasible. The incidence of infection following UKA is low (<1%), and we wished to study specifically acute infections, thus only 19 cases were recorded out of 3225 operations. The retrospective nature leads to the possibility of incomplete data or loss to FU. Although full data were available for all patients recorded as infected, some patients may have been treated elsewhere. Historically, success in treating PJI has been recorded as a percentage free of infection, but there is increasing focus on functional outcomes, and the nature of this study means that this aspect has not been addressed.

## Conclusion

The present study showed a rate of success of 84.2% with a single DAIR in infected UKAs, and overall survivorship free from all-cause reoperation of 78.95%. DAIR had higher success rates compared to earlier published data for UKA, and significantly better survival than DAIR in TKA. Additionally, a second DAIR procedure was effective in patients who developed a re-infection. Given the more minimal nature of the UKA procedure, surgeons might wish to adopt a slightly different strategy to that in TKA infection, with more emphasis on the less invasive and potentially more successful DAIR procedure.

The Strengthening the Reporting of Observational Studies in Epidemiology (STROBE) guidelines were followed when preparing this manuscript [34].

## Data Availability

The anonymised datasets generated in this study are available from the corresponding author (NM) on reasonable request.

## Abbreviations

CRP: C-Reactive Protein
DAIR: Debridement Antibiotics and Implant Retention
ESR: Erythrocyte sedimentation rate
FU: Follow-up
ICM: International Consensus Meeting
IV: Intravenous
MicroL: Microlitres
mg/l: Milligrams/litre
MSIS: Musculoskeletal Infection Society
NJR: National Joint Registry
PJI: Periprosthetic Joint Infection
TKA: Total Knee Arthroplasty
UKA: Unicompartmental Knee Arthroplasty
UK: United Kingdom
WBC: White Blood Cells
